# Supplementary material for: Single cell plasticity and population coding stability in auditory thalamus upon associative learning
Source: Nat Commun. 2021 Apr 26;12:2438. doi: 10.1038/s41467-021-22421-8 (PMC8076296; doi:10.1038/s41467-021-22421-8)
Supplement: Supplementary file 1 — Supplementary Information [file 41467_2021_22421_MOESM1_ESM.pdf]

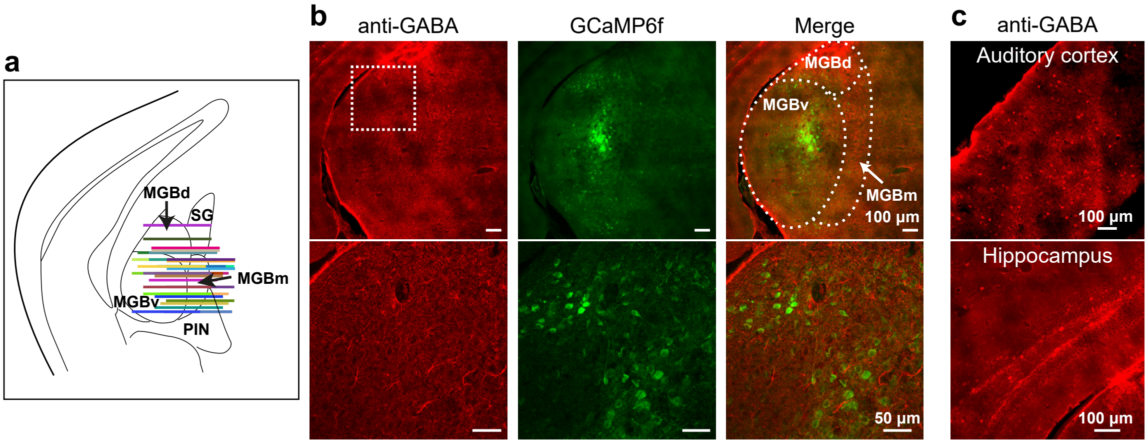

Supplementary Fig. 1: Imaging of excitatory cells in MGB.
a) GRIN lens front (horizontal lines) of all mice (N = 30). **b)** Immunohistochemistry for GABA in MGB (left), GCaMP6f-expressing cells (middle) and merge (right). Bottom row: higher magnification of square indicated in top left image. GABAergic fibres are distributed widely in MGB while GABAergic somata are mainly absent (N = 2 mice). **c)** GABA-positive somata in auditory cortex and hippocampus using the same antibody as in b (N = 2 mice).

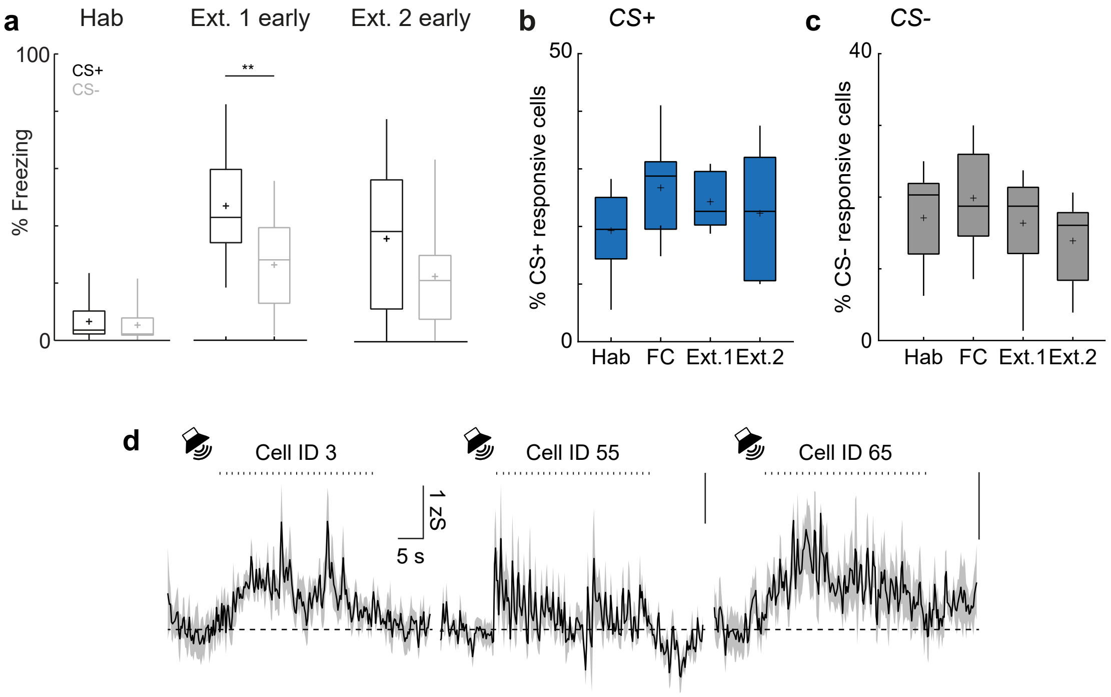

Supplementary Fig. 2: CS+ responsive cells, CS- freezing and CS- responsive cells.
a) Freezing to the CS+ and CS- during auditory fear conditioning. Ext. 1 early: CS+ 61 ± 6%, CS- 42 ± 8%, p = 0.0075, two-tailed Mann-Whitney test, N = 15 mice. Boxplots represent median, 2nd, 3rd quartile, minimum and maximum. Cross indicates mean. **b)** Percentage of CS+ responsive cells across all four imaging days. Hab = 19 ± 2%, FC = 26 ± 3%, Ext. 1 = 24 ± 2%, Ext. 2 = 22 ± 4% (Friedman test, p > 0.05, N = 9 mice). Boxplots represent median, 2nd, 3rd quartile, minimum and maximum. Cross indicates mean. **c)** Percentage of CS- responsive cells across all four imaging days. Hab = 17 ± 2%, FC = 20 ± 2%, Ext. 1 = 16 ± 2%, Ext. 2 = 14 ± 2% (Friedman test, p > 0.05, N = 9 mice). Boxplots represent median, 2nd, 3rd quartile, minimum and maximum. Cross indicates mean. **d)** CS- Ca^{2+} responses (mean ± s.e.m.) of three example MGB neurons during habituation.

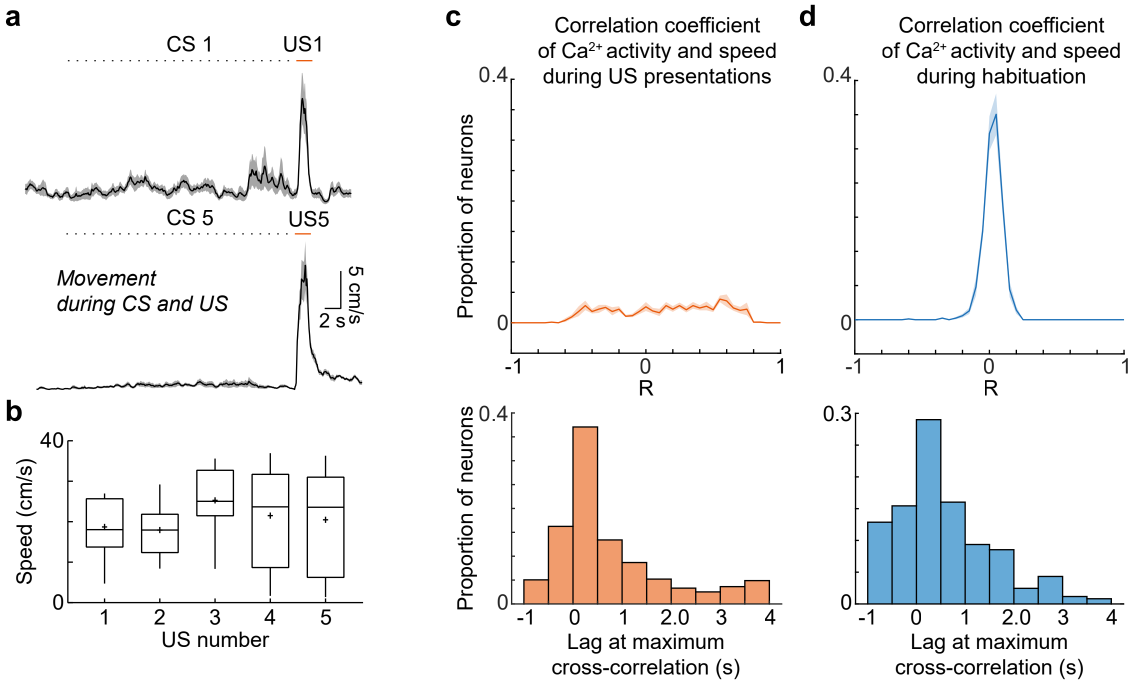

Supplementary Fig. 3: Correlation of neuronal activity and mouse movement.
a) Average speed ± s.e.m. of mouse during first CS+-US presentation (top) and last CS+-US presentation (bottom). **b)** Average speed across the 5 US presentations (Kruskal-Wallis test, $p > 0.05$, N = 9 mice). Boxplots represent median, 2nd, 3rd quartile, minimum and maximum. Cross indicates mean. **c)** Distribution of the maximum cross-correlation coefficients between Ca^{2+} activity of individual neurons and the mouse's speed during the footshock US (n = 855 neurons from N = 9 mice, top, data presented as mean ± s.e.m.). Lag of maximum cross-correlation coefficient (bottom). **d)** Distribution of the maximum cross-correlation coefficients between Ca^{2+} activity of individual neurons and the mouse's speed during the habituation session outside of CS periods (n = 855 neurons from N = 9 mice, top, data presented as mean ± s.e.m.). Lag of maximum cross-correlation coefficient (bottom).

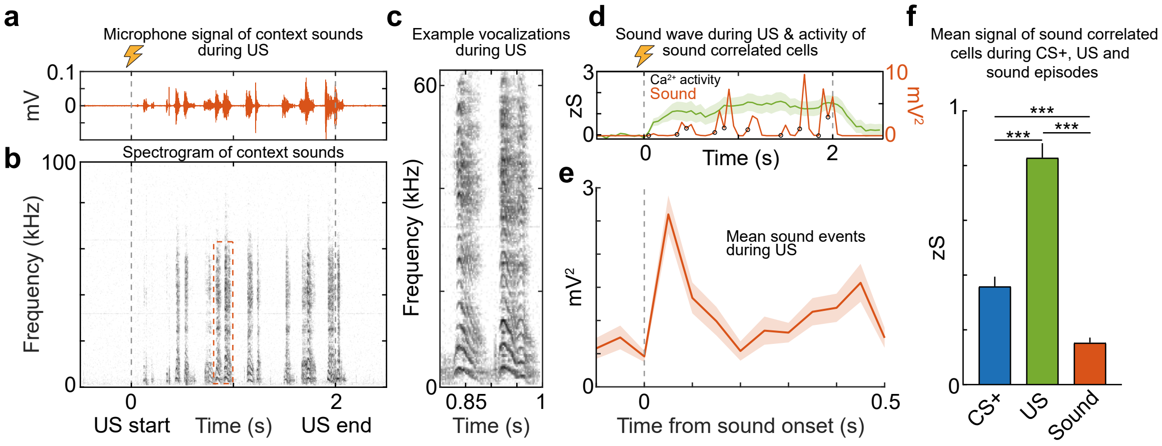

Supplementary Fig. 4: Ca^{2+} activity of MGB neurons is weakly correlated with self-evoked sounds during the US.
a) Recorded sounds in the conditioning context during the two second US shock. **b)** Frequency spectrogram of the sound wave in a. **c)** Example low-frequency vocalizations during the US from the outlined box in b. **d)** Downsampled squared sound wave from a and the mean Ca^{2+} activity ± s.e.m. of cells which exhibited a cross-correlated Ca^{2+} response. Circles indicate the onset of sound events. **e)** Mean (± s.e.m.) of onset-aligned detected sound events during all US presentations (n = 15 US presentations and 135 sound events from N = 3 mice, downsampled to match 20 Hz imaging frequency). **f)** Summary statistics of the mean Ca^{2+} response in Fig. 2k (0-300 ms) indicate that US and CS responses are stronger than self-evoked sound responses (Kruskal-Wallis test with Dunn's multiple comparisons test, all $p < 0.001$, N = 3 mice, data presented as mean values ± s.e.m.).

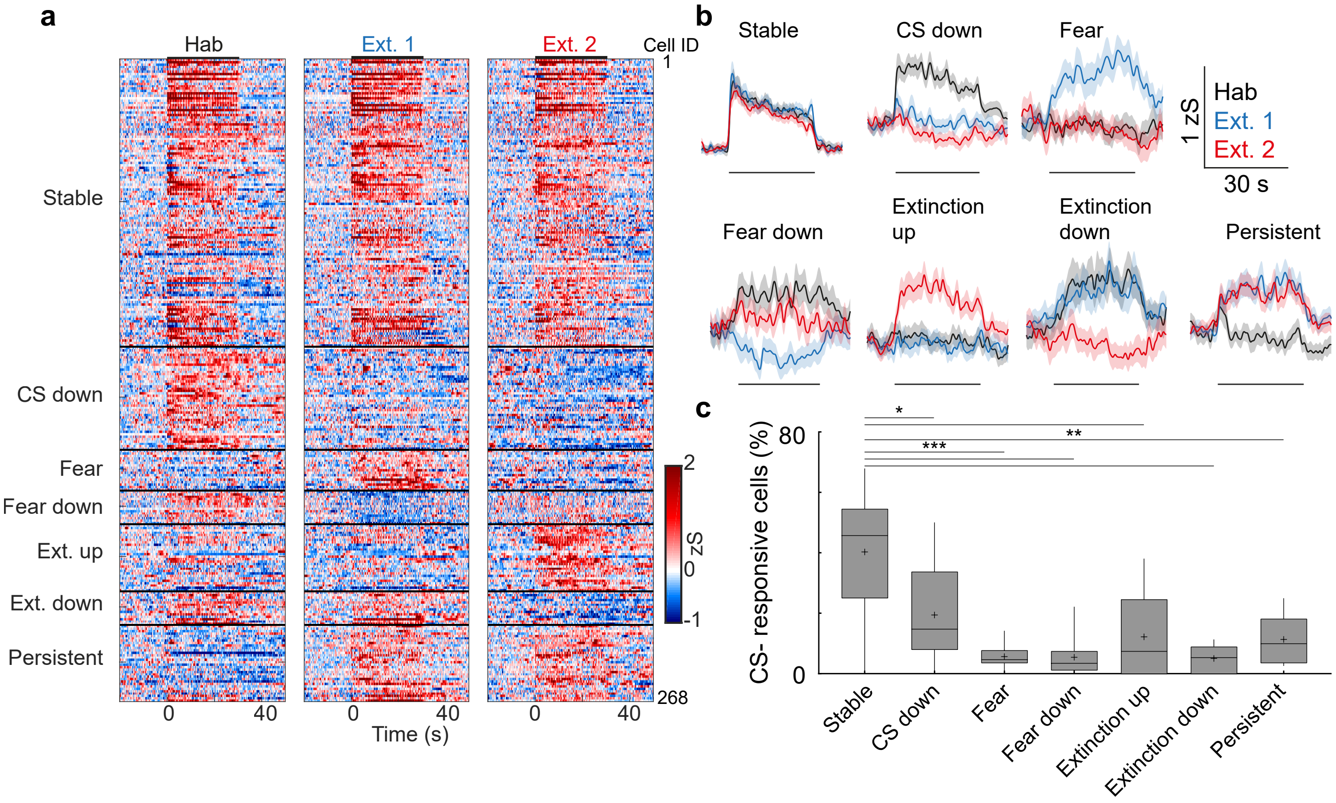

Supplementary Fig. 5: US and CS- response plasticity of individual MGB neurons.
a) Response map of all CS-responsive neurons on the habituation, extinction 1 and extinction 2 days sorted by response type (n = 268 cells, N = 9 mice). **b)** Average Ca^{2+} traces of the different CS- response groups on the habituation (black), extinction 1 (blue) and extinction 2 (red) days. Traces represent mean ± s.e.m.. **c)** Quantification of CS- responses (Friedman test, $p < 0.001$, followed by Dunn's multiple comparisons test, stable vs CS down p = 0.036, stable vs fear $p < 0.0001$, stable vs fear down $p <0.0001$, stable vs. extinction up p = 0.0014; stable vs extinction down, $p < 0.01$; stable vs persistent p = 0.0009, N = 9 mice). Boxplots represent median, 2nd, 3rd quartile, minimum and maximum. Cross indicates mean. *, **, *** indicate p-values smaller than 0.05, 0.01 and 0.001, respectively.

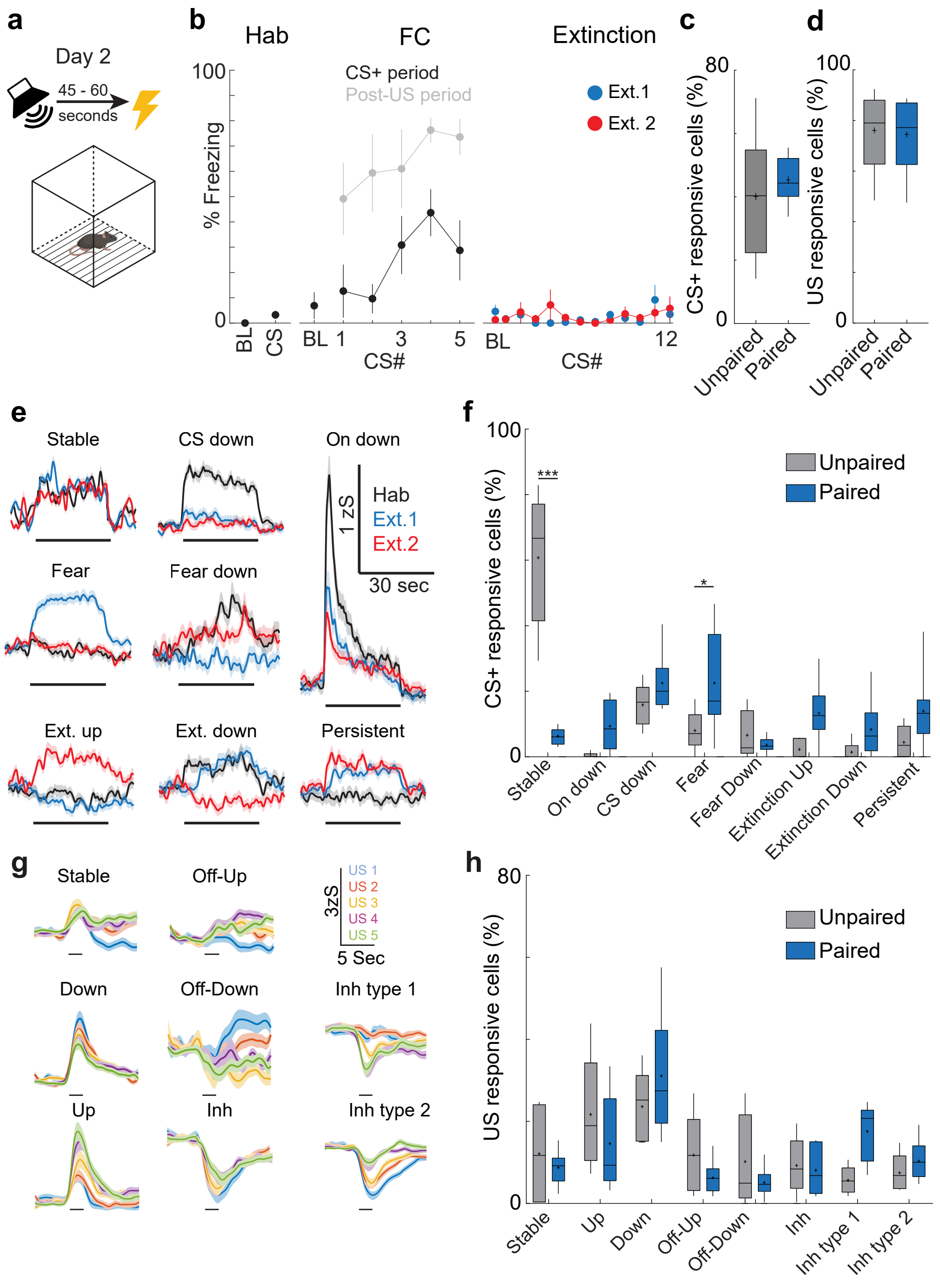

Supplementary Fig. 6: CS+ responses are mostly stable across days upon unpaired conditioning. **a)** Schematic of unpaired conditioning paradigm. The CS+ and US are separated by random intervals between 45 to 60 s. **b)** Mean ± s.e.m. trial-by-trial freezing to CS+ or the post-US period (grey) on the habituation, fear conditioning and extinction days (N = 5 mice). **c)** Proportion of CS+ responsive neurons in fear conditioned (paired, 45 ± 2%, N = 9 mice, see Fig. 2) and unpaired conditioned animals (unpaired, 40 ± 8%, N = 5 mice, two-tailed Mann-Whitney test, p = 0.529). Boxplots represent median, 2nd, 3rd quartile, minimum and maximum. Cross indicates mean. **d)** Percentage of US responsive cells in the paired (74 ± 5%, N = 9) and unpaired (76 ± 7%, N = 5) group (two-tailed Mann-Whitney test, p = 0.898). Boxplots represent median, 2nd, 3rd quartile, minimum and maximum. Cross indicates mean. **e)** Diversity of mean CS+ responses ± s.e.m. across days in the group of plastic neurons upon unpaired conditioning. **f)** Quantification of the proportion of neurons within each CS+ plasticity clusters for fear conditioned and unpaired animals (2-way ANOVA, p < 0.05 followed by Sidak's multiple comparisons test; stable: paired vs unpaired, p < 0.001, Fear neurons: paired vs unpaired, p = 0.0303; N = 9 mice for fear conditioning (see Fig. 2) and N = 5 mice for unpaired conditioning). Boxplots represent median, 2nd, 3rd quartile, minimum and maximum. Cross indicates mean. **g)** Diversity of mean US responses ± s.e.m. across trials upon unpaired conditioning (traces from N = 5 mice). **h)** Quantification of the proportion of US clusters for fear conditioned and unpaired animals (2-way ANOVA, p < 0.05, followed by Sidak's multiple comparisons test p > 0.05, N = 5 mice for unpaired conditioning and N = 9 for paired conditioning). Boxplots represent median, 2nd, 3rd quartile, minimum and maximum. Cross indicates mean. * indicate p-values smaller than 0.05.

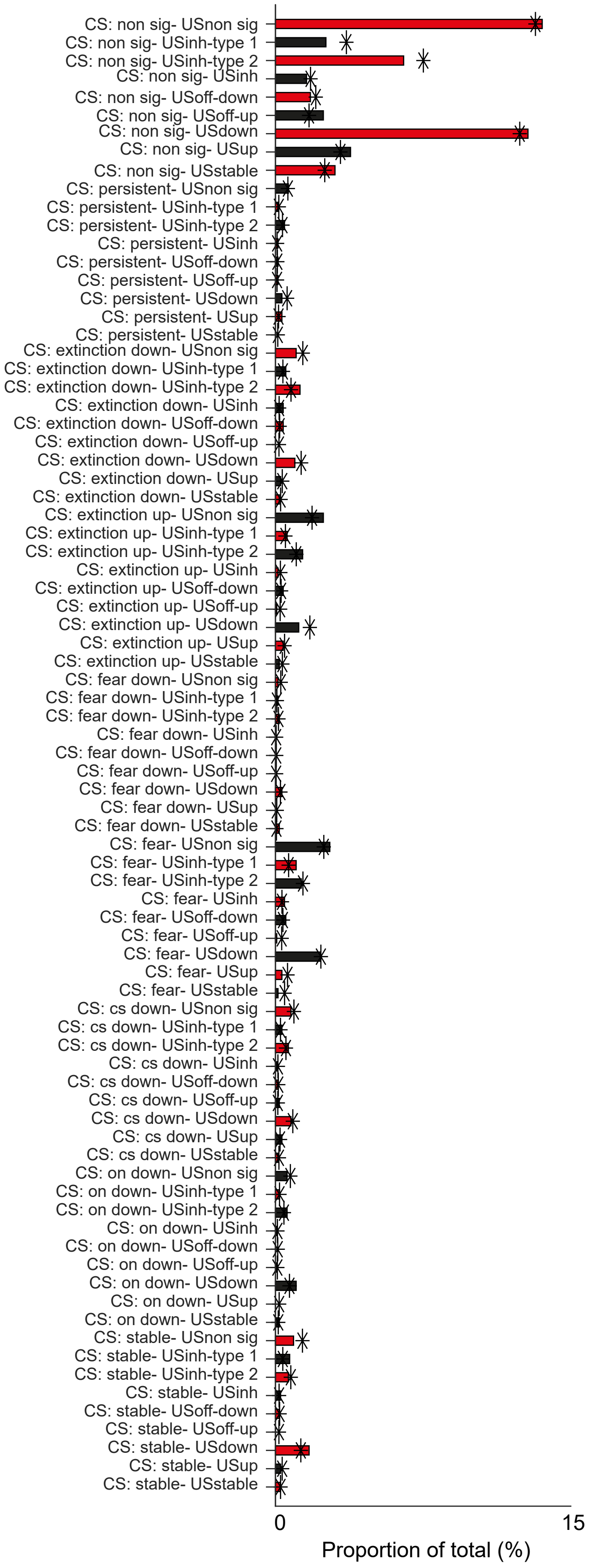

Supplementary Fig. 7: CS response type is not predictive of US plasticity.
Proportion of overlapping subgroups of CS+ and US responsive cells (N = 9 mice). *indicates chance levels of finding overlapping groups based on the product of the proportions of individual subgroups.

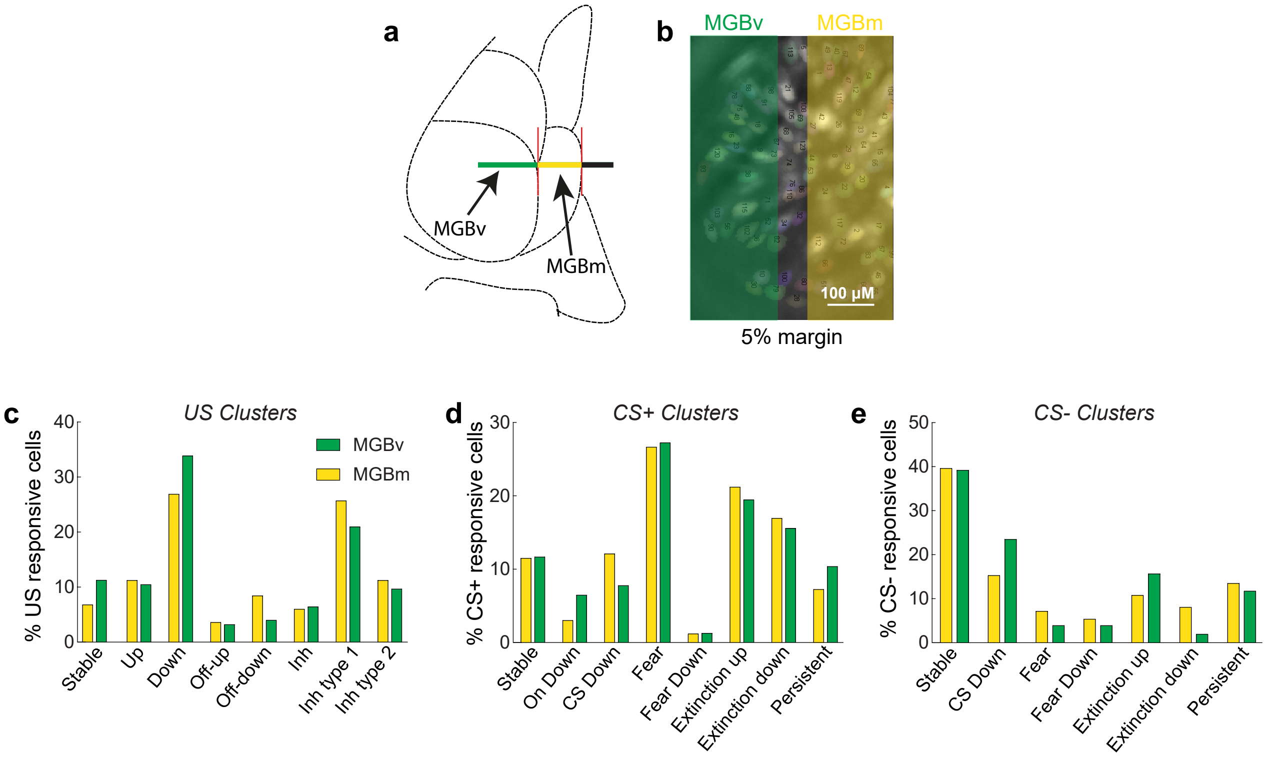

Supplementary Fig. 8: Similar CS and US plasticity types between MGB subdivisions.
a) Schematic of GRIN lens location above different MGB subdivisions. **b)** Field of view of a divided into subregions. Done for all mice with a GRIN lens above both MGBv and MGBm (N = 5 mice). **c-e)** Proportion of cells belonging to the different US plasticity types (c), CS+ plasticity types (d) and CS- plasticity types (e) for each subdivision.

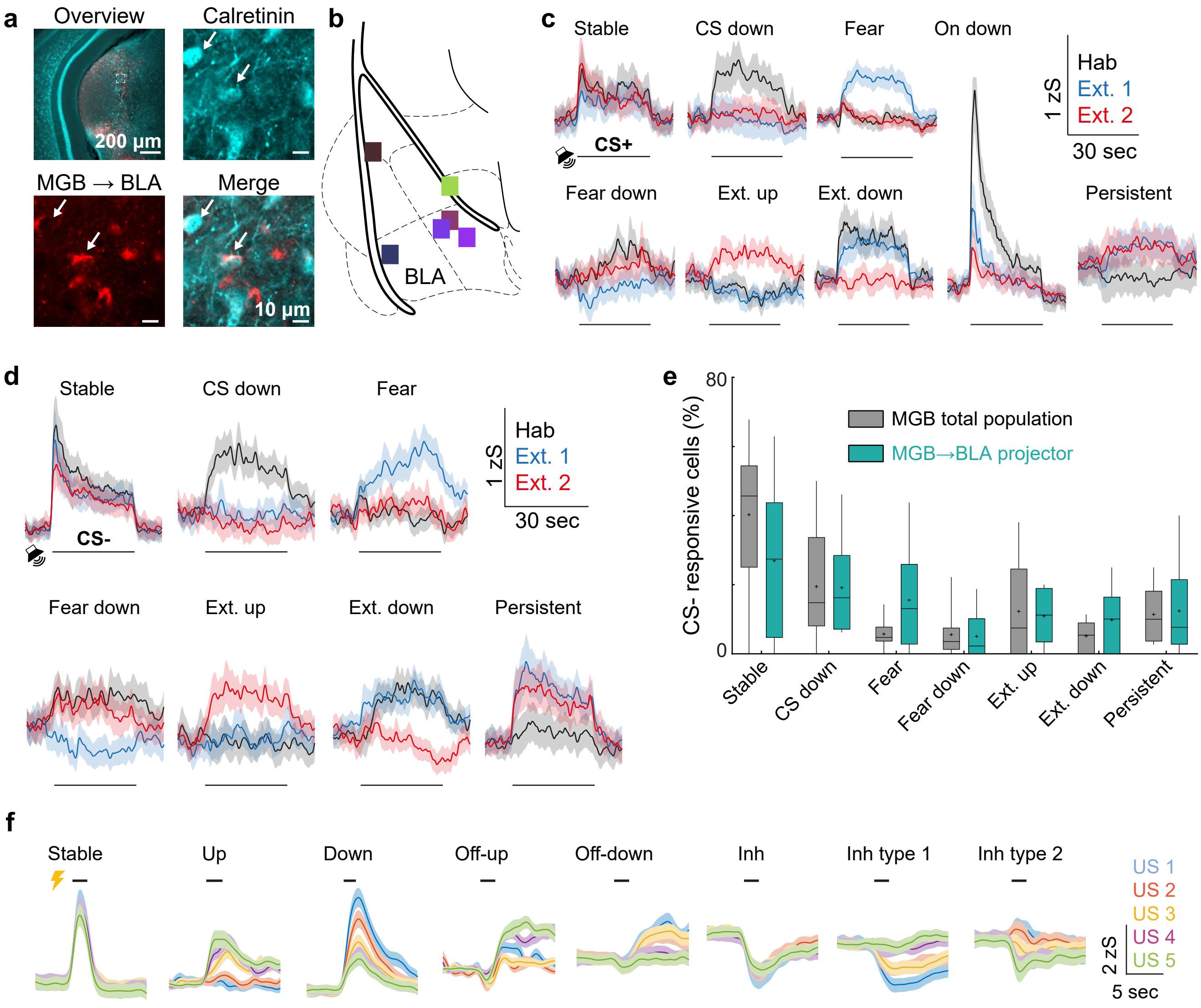

Supplementary Fig. 9: CS and US plasticity of MGB→BLA projection neurons.
a) CTB labelling of MGB→BLA projection neurons and immunohistochemistry for calretinin. Arrows indicate double-positive cells (N = 6 mice). **b)** Center of virus injection sites of mice included in MGB→BLA projection neuron imaging (N = 6 mice). **c)** Average Ca^{2+} traces of CS+ plasticity subgroups of MGB→BLA projection neurons. Mean ± s.e.m.. Horizontal lines indicate CS+ period. **d)** Average Ca^{2+} traces of CS- plasticity subgroups of MGB→BLA projection neurons. Mean ± s.e.m.. Horizontal lines indicate CS- period. **e)** Quantification of CS+ plasticity subgroups in the total MGB population (N = 9 mice) and MGB→BLA projection neurons (N = 5 mice). 2-way ANOVA, $p > 0.05$ Boxplots represent median, 2nd, 3rd quartile, minimum and maximum. Cross indicates mean. **f)** Average Ca^{2+} traces of US plasticity subgroups of MGB→BLA projection neurons. Mean ± s.e.m.. Horizontal lines indicate US period.

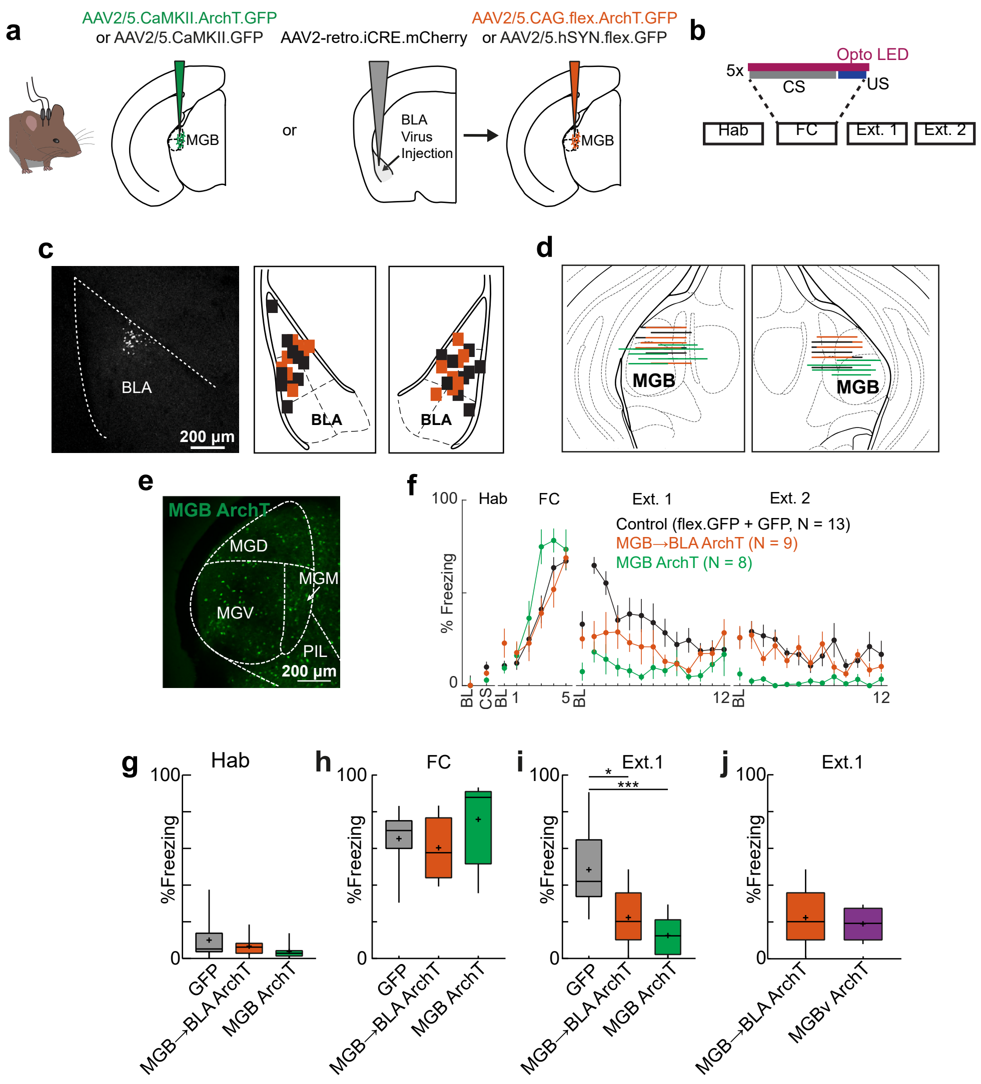

Supplementary Fig. 10: Optogenetic inhibition of MGB as well as MGB→BLA projection neurons supresses consolidation of fear learning.

a) Viral expression strategy for optogenetic manipulation of MGB neurons and MGB→BLA projecting neurons. **b)** Schematic of optogenetic manipulation on the fear conditioning day. **c)** Confocal microscopy image of injetion site marker (latex beads) location in the basolateral amygdala (BLA) of one example mouse (left). Replicated for all animals shown on right. Center of rAAV2-retro.icre injection sites in BLA for all animals (N = 19 mice, right). **d)** Optical fibre fronts in MGB for the MGB→BLA group (orange, N = 9 mice), the MGB group (green, N = 8 mice) and and the GFP control group (black, N = 13). **e)** Example of CaMKII-driven ArchT expression in MGB population. This was replicated in all animals that underwent inhibition of MGB neurons (N = 8 mice) **f)** Mean +/- s.e.m. freezing across the 4-day fear conditioning paradigm for MGB→BLA ArchT (orange, N = 9 mice), GFP (black, N = 13 mice) and MGB ArchT mice (green, N = 8). **g)** Quantification of freezing on the habituation day (Kruskal-Wallis test, $p > 0.05$) MGB→ BLA ArchT (orange, N = 9 mice), GFP (black, N = 13 mice) and MGB ArchT mice (green, N = 8). Boxplots represent median, 2nd, 3rd quartile, minimum and maximum. Cross indicates mean. **h)** Average freezing of GFP (N = 13 mice) and ArchT-expressing animals at the end of the fear conditioning paradigm (freezing to last two CS, control: 65 ± 4% freezing, MGB→BLA ArchT (N = 9 mice): 60 ± 6% freezing, total MGB ArchT (N = 8 mice): 75 ± 8% freezing; $p > 0.05$, Kruskal-Wallis test). Boxplots represent median, 2nd, 3rd quartile, minimum and maximum. Cross indicates mean. **i)** Average freezing of control and ArchT-expressing animals upon fear recall during early extinction 1 (Ext. 1, freezing during first four CS+, control: 48 ± 6%, N = 13 mice, MGB→BLA ArchT: 22 ± 5%, MGB ArchT: 13 ± 4%, N = 8 mice, Kruskal-Wallis test, $p < 0.05$, followed by Dunn's multiple comparisons test; Control vs MGB→BLA ArchT p = 0.033, Control vs MGB ArchT p = 0.0005, MGB→BLA ArchT vs MGB ArchT p = 0.68). Boxplots represent median, 2nd, 3rd quartile, minimum and maximum. Cross indicates mean. **j)** Average freezing of MGB→BLA ArchT and MGBv ArchT animals upon fear recall during early extinction 1 (Ext. 1, freezing during first four CS, MGB→BLA ArchT: 22 ± 5%, N = 9, MGBv ArchT: 19 ± 5%, N = 4). Boxplots represent median, 2nd, 3rd quartile, minimum and maximum. Cross indicates mean. *, *** indicate p-values smaller than 0.05 and 0.001, respectively.

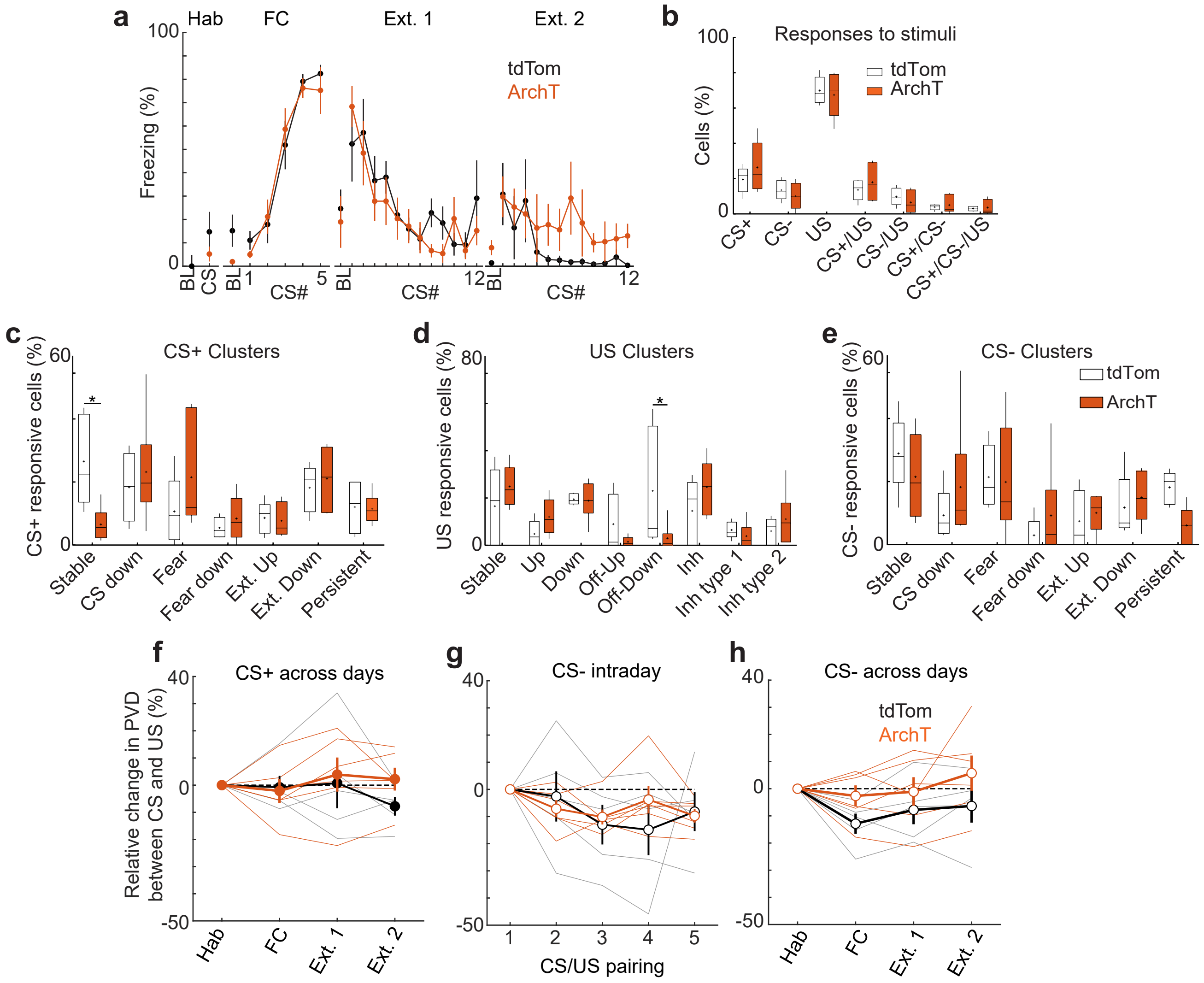

Supplementary Fig. 11: Neuronal responses and population coding during all-optical activity recording of MGB and inhibition of MGB→BLA neurons.
a) Mean ± s.e.m. freezing to the CS+ during the FC paradigm (MGB→BLA ArchT: N = 6 mice; tdTom: N = 5 mice). **b)** Proportion of cells responding to the CS+, CS-, US and combinations thereof (MGB→BLA ArchT: N = 6 mice; tdTom: N = 5 mice). Boxplots represent median, 2nd, 3rd quartile, minimum and maximum. Cross indicates mean. **c)** Quantification of CS+ plasticity subgroups in the Control (N = 5 mice) and ArchT neurons (N = 6 mice) (2-way ANOVA followed by Sidak's post hoc test, $p < 0.005$; Stable $p = 0.013$). Boxplots represent median, 2nd, 3rd quartile, minimum and maximum. Cross indicates mean. **d)** Quantification of US plasticity subgroups in the Control (N = 5 mice) and ArchT neurons (N = 6 mice; 2-way ANOVA followed by Sidak's post hoc test, $p < 0.005$; Off-Down, $p = 0.197$). Boxplots represent median, 2nd, 3rd quartile, minimum and maximum. Cross indicates mean. **e)** Quantification of CS- plasticity subgroups in the Control (N = 5 mice) and ArchT neurons (N = 6 mice). Boxplots represent median, 2nd, 3rd quartile, minimum and maximum. Cross indicates mean. **f-h)** Relative change in population vector distance between the CS+ (f) or CS- (j-h) and the US within the fear conditioning session (j) or across the individual days of the behavioural paradigm (j, h) (MGB→BLA ArchT: N = 6 mice; tdTom: N = 5 mice, data presented as mean values ± s.e.m.). * indicate p-values smaller than 0.05.

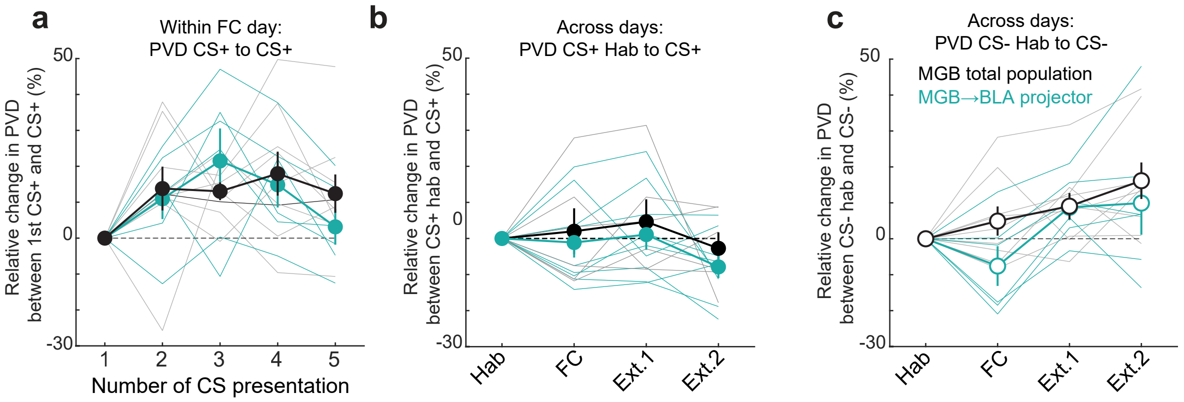

Supplementary Fig. 12: CS+ to CS+ and CS- to CS- population vector distances.
a) Relative change in PVD between first CS+ and subsequent CS+ presentations during the fear conditioning paradigm (data presented as mean values ± s.e.m.). **b)** Relative change in PVD between the CS+ on the habituation day and the CS+ presentations on subsequent days (data presented as mean values ± s.e.m.). **c)** Relative change in PVD between the CS- on the habituation day and CS- presentations on subsequent days. MGB total population: N = 9 mice, MGB-->BLA projectors: N = 6 mice (data presented as mean values ± s.e.m.).

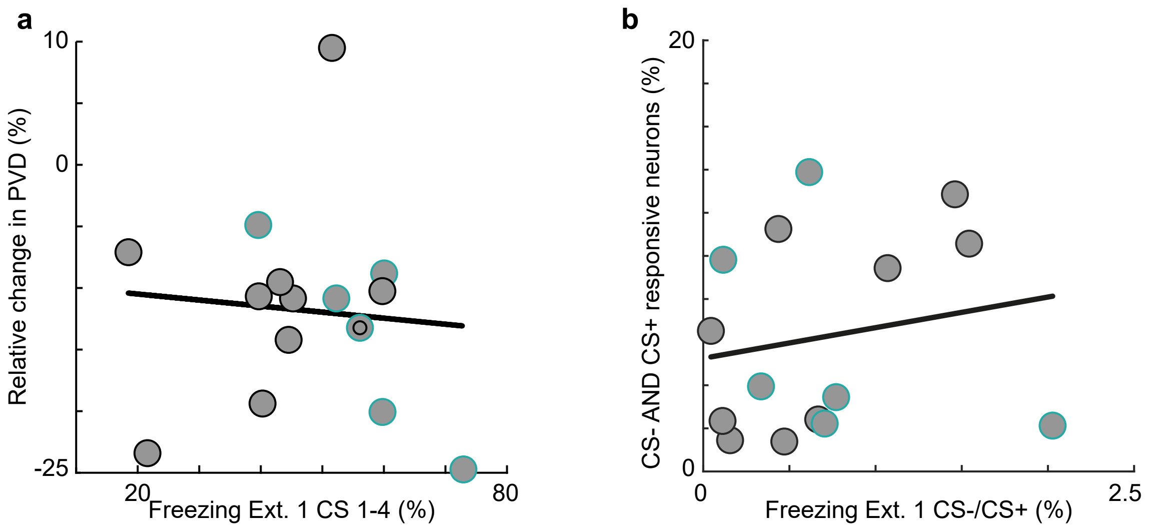

Supplementary Fig. 13: Correlation of relative change in PVD as well as proportions of CS plastic neurons with fear learning or fear generalization.

a) Scatter plot of change in population vector distance (ΔPVD) and pre-extinction freezing behaviour for N = 15 mice. Black dots: Total MGB population. Cyan dots: MGB→BLA projector population. Line: Linear regression (R^2 = -0.07, $p > 0.05$). **b)** Scatter plot of the proportion of neurons that are responsive to both, the CS- and the CS+ vs. the generalization of freezing during early extinction on day 1. Black stroke: total MGB population. Cyan stroke: MGB→BLA projecting population. Line: linear regression (R^2 = 0.03, $p > 0.05$). N = 15 mice.

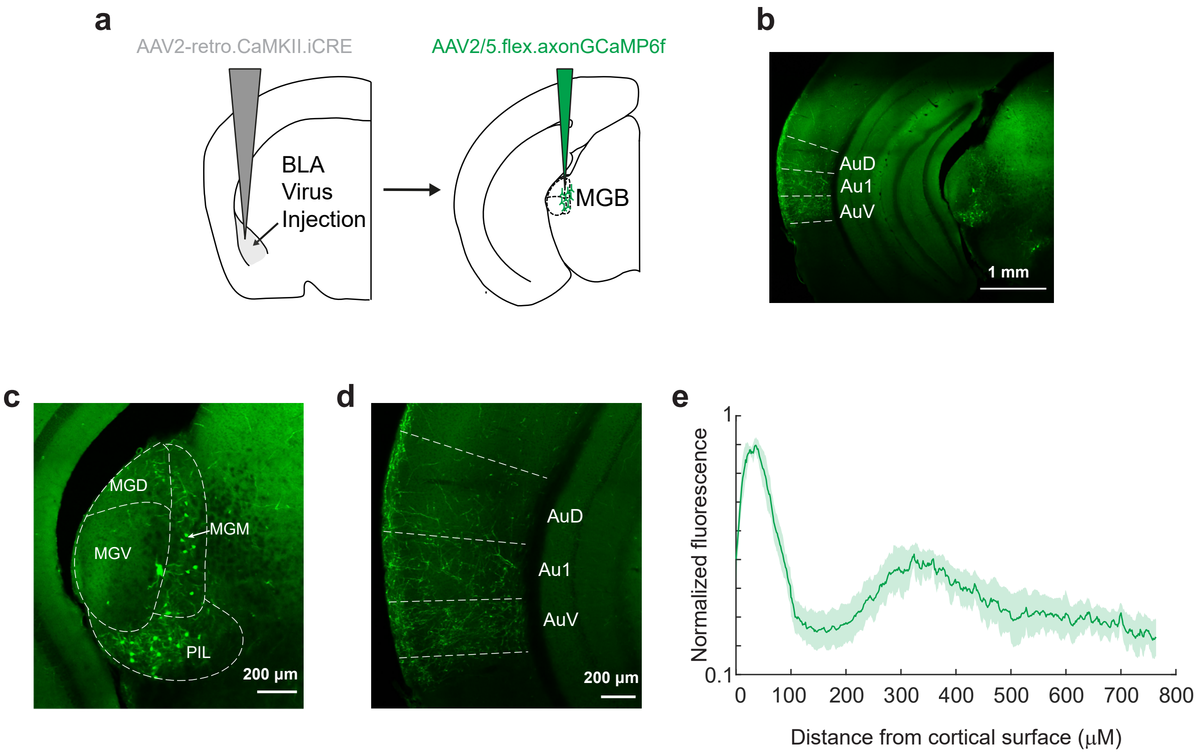

Supplementary Fig. 14: MGB→BLA neurons send collaterals to auditory cortex.
a) Schematic of tracing approach. **b)** Overview confocal microscopy image of stained neurons and axons. **c)** Magnification of MGB from b. **d)** Magnification of auditory cortex from b. **e)** Average fluorescence ± s.e.m. across auditory cortex (n = 3 slices / mouse, N = 3 mice).

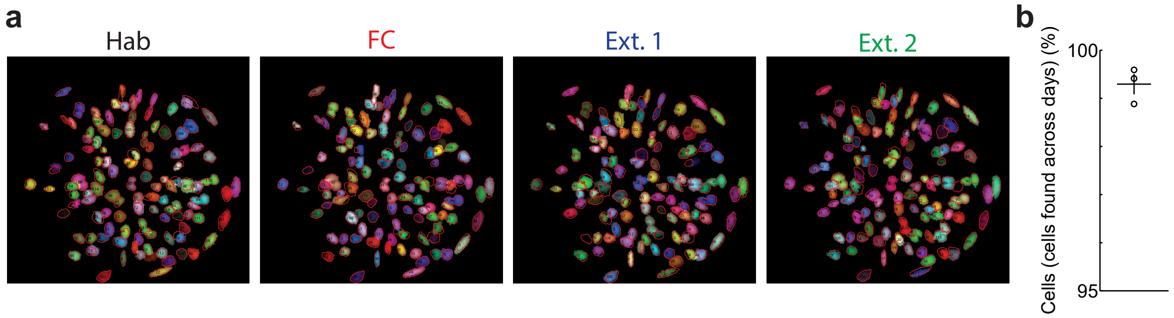

Supplementary Fig. 15: Stability of neuronal detection across days.
a) Cell maps for independent cell sorting based on individual days. Red outlines show ICs based on concatenated across day cell sorting for one example mouse. **b)** Percentage of cells found based on across day cell sorting which were also found based on each individual day cell sorting (99.3% ± 0.2, N = 3 mice, data presented as mean values ± s.e.m.).

Correlation neuronal activity and CS+ freezing

**CS+ clusters/CS+ Freezing**	**Stable**	**On down**	**CS down**	**Fear**	**Fear Down**	**X up**	**X down**	**Persistent**
X1 early	R^2 = 0.0667	R^2 = 0.0004	R^2 = 0.396	R^2 = 0.0044	R^2 = 0.0040	R^2 = 0.0001	R^2 = 0.0097	R^2 = 0.2338
X2 late	R^2 = 0.0013	R^2 = 0.0546	R^2 = 0.0011	R^2 = 0.0521	R^2 = 0.0866	R^2 = 0.0684	R^2 = 0.0036	R^2 = 0.0361

**US clusters/CS+ Freezing**	**Stable**	**Up**	**Down**	**Off-up**	**Off down**	**Inh**	**Inh type 1**	**Inh type 2**
X1 early	R^2 = 0.0078	R^2 = 0.2738	R^2 = 0.0041	R^2 = 0.2566	R^2 = 0.2178	R^2 = 0.0064	R^2 = 0.0094	R^2 = 0.0147
X2 late	R^2 = 0.0031	R^2 = 0.0165	R^2 = 0.0306	R^2 = 0.0258	R^2 = 0.0248	R^2 = 0.00305	R^2 = 0.0087	R^2 = 0.0119

**CS- clusters/CS+ Freezing**	**Stable**	**CS down**	**Fear**	**Fear Down**	**X up**	**X down**	**Persistent**
X1 early	R^2 = 0.0920	R^2 = 0.2272	R^2 = 0.0010	R^2 = 0.0627	R^2 = 0.0634	R^2 = 0.00390	R^2 = 0.0770
X2 late	R^2 = 0.0309	R^2 = 0.0147	R^2 = 0.0238	R^2 = 0.1769	R^2 = 0.0581	R^2 = 0.0280	R^2 = 0.3992

Correlation neuronal activity and CS- /CS+ freezing

**CS+ clusters**	**Stable**	**On down**	**CS down**	**Fear**	**Fear Down**	**X up**	**X down**	**Persistent**
CS-/CS+ X1 early	R^2 = 0.0080	R^2 = 0.1112	R^2 = 0.0156	R^2 = 0.0025	R^2 = 0.0134	R^2 = 0.0027	R^2 = 0.0938	R^2 = 0.0399

**US clusters**	**Stable**	**Up**	**Down**	**Off-up**	**Off down**	**Inh**	**Inh type 1**	**Inh type 2**
CS-/CS+ X1 early	R^2 = 0.0113	R^2 = 0.1444	R^2 = 00059	R^2 = 0.4772	R^2 = 0.1439	R^2 = 0.0408	R^2 = 0.0128	R^2 = 0.0347

**CS- Clusters**	**Stable**	**CS down**	**Fear**	**Fear Down**	**X up**	**X down**	**Persistent**
CS-/CS+ X1 early	R^2 = 0.0004	R^2 = 0.4702	R^2 = 0.0010	R^2 = 0.0056	R^2 = 0.1256	R^2 = 0.0004	R^2 = 0.1068

Table 1: Correlation between neuronal activity and freezing.
Significant correlations are labelled in red ($p < 0.01$) and blue ($p < 0.05$) (F-test of linear model).

Table 1
Taylor et al.
