# Supplementary material for: Single cell plasticity and population coding stability in auditory thalamus upon associative learning
Source: Nat Commun. 2021 Apr 26;12:2438. doi: 10.1038/s41467-021-22421-8 (PMC8076296; doi:10.1038/s41467-021-22421-8)
Supplement: Supplementary file 2 — Reporting Summary [file 41467_2021_22421_MOESM2_ESM.pdf]

nature research

Corresponding author(s): Jan Gründemann

Last updated by author(s): Feb 17, 2021

Reporting Summary

Nature Research wishes to improve the reproducibility of the work that we publish. This form provides structure for consistency and transparency in reporting. For further information on Nature Research policies, see Authors & Referees and the Editorial Policy Checklist .

Statistics

For all statistical analyses, confirm that the following items are present in the figure legend, table legend, main text, or Methods section.

n/a	Confirmed	
☐	☒	The exact sample size (*n*) for each experimental group/condition, given as a discrete number and unit of measurement
☐	☒	A statement on whether measurements were taken from distinct samples or whether the same sample was measured repeatedly
☐	☒	The statistical test(s) used AND whether they are one- or two-sided<br>*Only common tests should be described solely by name; describe more complex techniques in the Methods section.*
☐	☒	A description of all covariates tested
☐	☒	A description of any assumptions or corrections, such as tests of normality and adjustment for multiple comparisons
☐	☒	A full description of the statistical parameters including central tendency (e.g. means) or other basic estimates (e.g. regression coefficient) AND variation (e.g. standard deviation) or associated estimates of uncertainty (e.g. confidence intervals)
☐	☒	For null hypothesis testing, the test statistic (e.g. *F*, *t*, *r*) with confidence intervals, effect sizes, degrees of freedom and *P* value noted<br>*Give P values as exact values whenever suitable.*
☒	☐	For Bayesian analysis, information on the choice of priors and Markov chain Monte Carlo settings
☒	☐	For hierarchical and complex designs, identification of the appropriate level for tests and full reporting of outcomes
☒	☐	Estimates of effect sizes (e.g. Cohen's *d*, Pearson's *r*), indicating how they were calculated

Our web collection on statistics for biologists contains articles on many of the points above.

Software and code

Policy information about availability of computer code

Data collection	Behaviour analysis: EthoVision version 14.0 and version 15.0, Cineplex 3.4.1<br>Calcium imaging: nVista Software Version: 2.0.4 and nVoke software 2.1<br>Confocal microscope: Zen 2.1 (black, Zeiss).
Data analysis	Matlab R2019b (Mathworks) and custom written code<br>ImageJ 2.0.0-rc-49/1.51a (NIH)<br>Prism 8 (GraphPad)

For manuscripts utilizing custom algorithms or software that are central to the research but not yet described in published literature, software must be made available to editors/reviewers. We strongly encourage code deposition in a community repository (e.g. GitHub). See the Nature Research guidelines for submitting code & software for further information.

Data

Policy information about availability of data

All manuscripts must include a data availability statement. This statement should provide the following information, where applicable:
- Accession codes, unique identifiers, or web links for publicly available datasets
- A list of figures that have associated raw data
- A description of any restrictions on data availability

The source data underlying the main figures and Supplementary information are available as Source Data file.

Field-specific reporting

Please select the one below that is the best fit for your research. If you are not sure, read the appropriate sections before making your selection.

- [x] Life sciences
- [ ] Behavioural & social sciences
- [ ] Ecological, evolutionary & environmental sciences

For a reference copy of the document with all sections, see nature.com/documents/nr-reporting-summary-flat.pdf

Life sciences study design

All studies must disclose on these points even when the disclosure is negative.

Sample size

All sample sizes were coherent with standard practices in the field and previous publications in Nature journals (Penzo et al., 2015, Nature or Barsy et al., 2020, Nature Neuroscience, Krabbe et al., 2019, Nature Neuroscience, Grewe et al., 2017, Nature).

Data exclusions

The only exclusion criterion was post-hoc validation of wrongly targeted viral injection and GRIN lens or fiber placements. E.g. Virus injection outside of the amygdala or medial geniculate body.

Replication

The exact number of repetitions (individual data points from separate cells and/or animals) are indicated in figures and legens. Averaging across multiple trials per cell/animal is indicated where apllicable and n/N numbers always refer to data from individual cells/animals, no samples were measured repeatedly for statistical analysis. All attempts of replication were successful across animals and reflected in the numbers reported.

Multiple rounds of experimentation were required, i.e., from multiple mice, which were averaged for the presented datasets. Data was acquired from mice from multiple litters, and responses from individual cells were collected from at least three mice per group. No results were included that were not observed in multiple animals. No issues were identified in reproducing any of the reported findings within groups. However, we did not use replication per se (as in multiple separate cohorts of several subjects).

Randomization

Mice of the same age and sex (male) were used for the imaging and behavioural experiments in this study (8-11 weeks at the start of the experiment). Litter mates were randomly assigned to the experimental groups without predetermined criteria.

Blinding

During the optogenetic experiments and analysis, the experimenter was blind to the experimental condition.
For miniscope imaging, blinding was not necessary as there were no experimental groups with different treatments. To classify different functional neuronal types, all cells from all animals were pooled together, clustered on the total population of neurons within this study and then reassigned to each animal to avoid bias.

Reporting for specific materials, systems and methods

We require information from authors about some types of materials, experimental systems and methods used in many studies. Here, indicate whether each material, system or method listed is relevant to your study. If you are not sure if a list item applies to your research, read the appropriate section before selecting a response.

Materials & experimental systems

n/a	Involved in the study	
[ ]	[x]	Antibodies
[x]	[ ]	Eukaryotic cell lines
[x]	[ ]	Palaeontology
[ ]	[x]	Animals and other organisms
[x]	[ ]	Human research participants
[x]	[ ]	Clinical data

Methods

n/a	Involved in the study	
[x]	[ ]	ChIP-seq
[x]	[ ]	Flow cytometry
[x]	[ ]	MRI-based neuroimaging

Antibodies

Antibodies used

Goat anti-Calretinin (Swant, CG1, lot 1§.1)
https://www.swant.com/pdfs/Goat_anti_calretinin_CG1.pdf

rabbit anti-NeuN (Abcam, ab177487, lot GR249899-66)
https://www.abcam.com/neun-antibody-epr12763-neuronal-marker-ab177487.html#top-300

rabbit anti-GABA (SigmaAldrich, A2052)
https://www.sigmaaldrich.com/catalog/product/sigma/a2052?lang=de&region=CH

Donkey anti-rabbit 555 (ThermoFisher, A31572, lot 1917920)
https://www.thermofisher.com/order/genome-database/generatePdf?productName=Rabbit%20IgG%20(H+L)%20Highly%20Cross-Adsorbed&assayType=PRANT&detailed=true&productId=A-31572

Donkey anti-rabbit 405 (Abcam, ab475651, lot GR3212998-1)
http://www.abcam.com/donkey-rabbit-igg-hl-alexa-fluor-405-ab175651.html

Donkey anti-goat 647 (ThermoFisher, A21447, lot 1841382)
https://www.thermofisher.com/order/genome-database/generatePdf?productName=Goat%20IgG%20(H+L)%20Cross-Adsorbed&assayType=PRANT&detailed=true&productId=A-21447

Validation	The antibodies have been validated and the relevant information can be found under Catalog numbers: CG1, ab177487, A2052, A31572, ab475651 and A21447, respectively (see webpage information above).

Animals and other organisms

Policy information about studies involving animals; ARRIVE guidelines recommended for reporting animal research

Laboratory animals	(Janvier) C57BL/6JRj adult male & female mice (8-11 weeks at the start of the experiment). Imaging and behavioural experiments were performed with male mice. Animals were housed on a 12-hour light / dark cycle at an ambient mean temperature and humidity of 22 C and 55 %, respectively. Food and water were provided ad libitum.
Wild animals	No wild animals were used in this study.
Field-collected samples	No field-collected samples were used in this study.
Ethics oversight	Animal experiments were done in accordance with institutional guidelines (University of Basel, Tierschutz) and were approved by the Cantonal Veterinary Office of Basel-Stadt, Switzerland.

Note that full information on the approval of the study protocol must also be provided in the manuscript.
